# Controlling Exchange Pathways in Dynamic Supramolecular Polymers by Controlling Defects

**DOI:** 10.1021/acsnano.1c01398

**Published:** 2021-09-02

**Authors:** Anna L. de Marco, Davide Bochicchio, Andrea Gardin, Giovanni Doni, Giovanni M. Pavan

**Affiliations:** † Department of Innovative Technologies, University of Applied Sciences and Arts of Southern Switzerland, Polo Universitario Lugano, Campus Est, Via la Santa 1, 6962 Lugano-Viganello, Switzerland; ‡ Department of Physics, Universit degli studi di Genova, Via Dodecaneso 33, 16100 Genova, Italy; § Department of Applied Science and Technology, Politecnico di Torino, Corso Duca degli Abruzzi 24, 10129 Torino, Italy

**Keywords:** supramolecular polymers, exchange pathways, defects, coarse-graining, molecular dynamics, unsupervised clustering

## Abstract

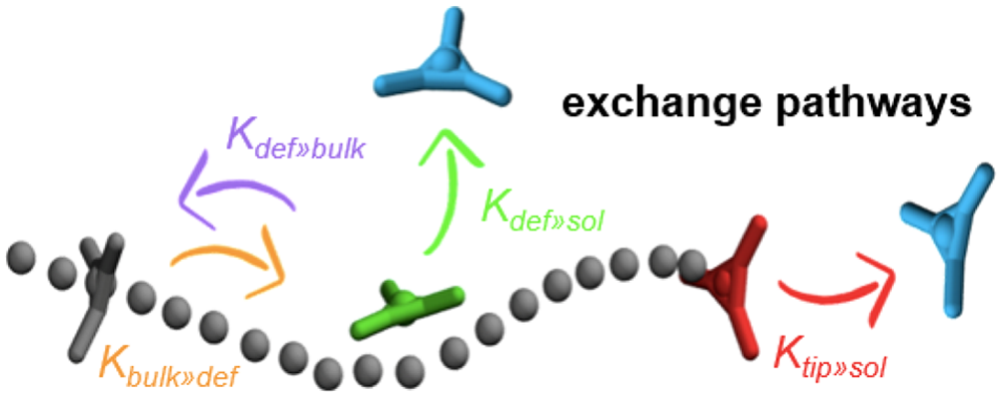

Supramolecular fibers composed of monomers that self-assemble directionally *via* noncovalent interactions are ubiquitous in nature, and of great interest in chemistry. In these structures, the constitutive monomers continuously exchange in-and-out the assembly according to a well-defined supramolecular equilibrium. However, unraveling the exchange pathways and their molecular determinants constitutes a nontrivial challenge. Here, we combine coarse-grained modeling, enhanced sampling, and machine learning to investigate the key factors controlling the monomer exchange pathways in synthetic supramolecular polymers having an intrinsic dynamic behavior. We demonstrate how the competition of directional *vs.* nondirectional interactions between the monomers controls the creation/annihilation of defects in the supramolecular polymers, from where monomers exchange proceeds. This competition determines the exchange pathway, dictating whether a fiber statistically swaps monomers from the tips or from all along its length. Finally, thanks to their generality, our models allow the investigation of molecular approaches to control the exchange pathways in these dynamic assemblies.

## Introduction

Supramolecular fibers, composed of fundamental building blocks that self-assemble directionally (1D) *via* noncovalent interactions, are ubiquitous in nature and play fundamental roles in living systems.^1,2^ Notable examples are cellular microtubules (MTs), dynamic assemblies composed of protein (tubulin) units whose dynamic polymerization and depolymerization are key for regulating the mechanical properties, motion, and differentiation of cells.^3,4^ The constitutive tubulin units bind to one end of the tubular assembly and detach from the other end, which makes MTs existing as dynamic entities, continuously growing on one side and shortening on the other. Such a specific exchange pathway for the tubulin building blocks allows, for example, for the generation of mechanical forces along the MTs, which are key in regulating many important functions, such as, *e.g.*, the migration, disassembly, and differentiation of the cells.^3,4^

Synthetic supramolecular polymers composed of monomeric units that self-assemble *via* noncovalent interactions (π–π stacking, hydrogen-bonding, shape recognition, solvophobic interactions, *etc.*) recently attracted great interest in the perspective of designing artificial materials possessing similar dynamic behaviors.^1,5−7^ Different from covalent polymers, and closer to biological assemblies (*e.g.*, MTs), in supramolecular polymers, the constitutive monomers exchange continuously in-and-out the assembly obeying a well-defined equilibrium.^8−10^ While the rate of this exchange is key to control bioinspired properties such as, for example, the ability of these materials to respond, adapt, or reconfigure in time in response to external stimuli,^11−14^ the pathway of the exchange is also of prime importance. In particular, learning how to customize the monomer structure in order to control the exchange pathways in the assembly would be appealing. For example, this would enable the rational design of supramolecular polymers that exchange monomers from the tips rather from their side surface (or vice versa), controlling their polymerization/depolymerization processes and the adaptivity of the assembly. Intriguingly, this would also allow one to design supramolecular entities that communicate with the external environment (exchanging monomers: molecular signaling/information) following to specific pathways, which are pre-encoded in the structure of their constitutive monomers, and which may change in specific ways in response to specific stimuli from the external environment.^10^

Despite important technical advancements,^12,15,16^ it is still prohibitive to experimentally monitor monomer exchange in supramolecular polymers at the necessary spatio-temporal resolution to unveil the monomer exchange pathways, as well as to study the involved mechanisms and determining factors. In particular, linking in an unambiguous way the dynamic behavior of the assembly to the structural features of the monomeric units, and to the monomer-monomer and monomer-environment interactions, most often remains a daunting task.

Computer simulations and molecular models can provide molecular-level information on the structure, thermodynamics, and dynamics of the assemblies that cannot be attained by the experiments.^17−24^ In particular, recently it has been demonstrated that the combination of coarse-grained (CG) models and metadynamics (MetaD) simulations allows studying the dynamics of a supramolecular polymer at submolecular resolution.^25−27^ This may provide useful molecular-level information on the factors that control the monomer exchange processes which are inaccessible to the experiments. The dynamic exchange of monomer between two identical fibers in the system can be schematized as divided into three steps: (i) monomer jumping out from a fiber, (ii) monomer diffusion in the solvent, and (iii) monomer adsorption onto another fiber. However, while step (ii) is mainly controlled by diffusion and it is most likely not influenced by tiny changes in the chemical structure of the monomers, and step (iii) is a barrier-free process which kinetics mainly depend on the concentration of monomers present in solution, step (i) is the most relevant step (worth of computational investigation) shaping the exchange pathway. Computational investigations of the dynamics of 1,3,5-benzenetricarboxamide (BTA) supramolecular polymers at high-resolution (∼5 Å) revealed that monomer exchange in the self-assembled fibers originates from defects in the supramolecular structure. These are less ordered, weaker, and more dynamic points in the assembly from which monomer exchange is most likely to proceed.^25^ In general, the role of defects in soft self-assembled materials is a topic of great current interest.^28^ In particular, such defects are vital for the dynamics of supramolecular polymers, as these constitute the source of monomer exchange events in the assembly. The generality of the tight relationship between defects and dynamics was proven in different types of supramolecular fibers.^14,21,26,27^ However, while the concept of defect is typically related to ordered and static structures such as crystals, identifying defects in such soft assembled fibers, where these are continuously and statistically created and repaired, is not trivial. Machine learning approaches are extremely useful to this end: these allow one to identify defects and to build a kinetic map for their formation and annihilation in such complex dynamic assemblies.^29^

**Figure 1:**
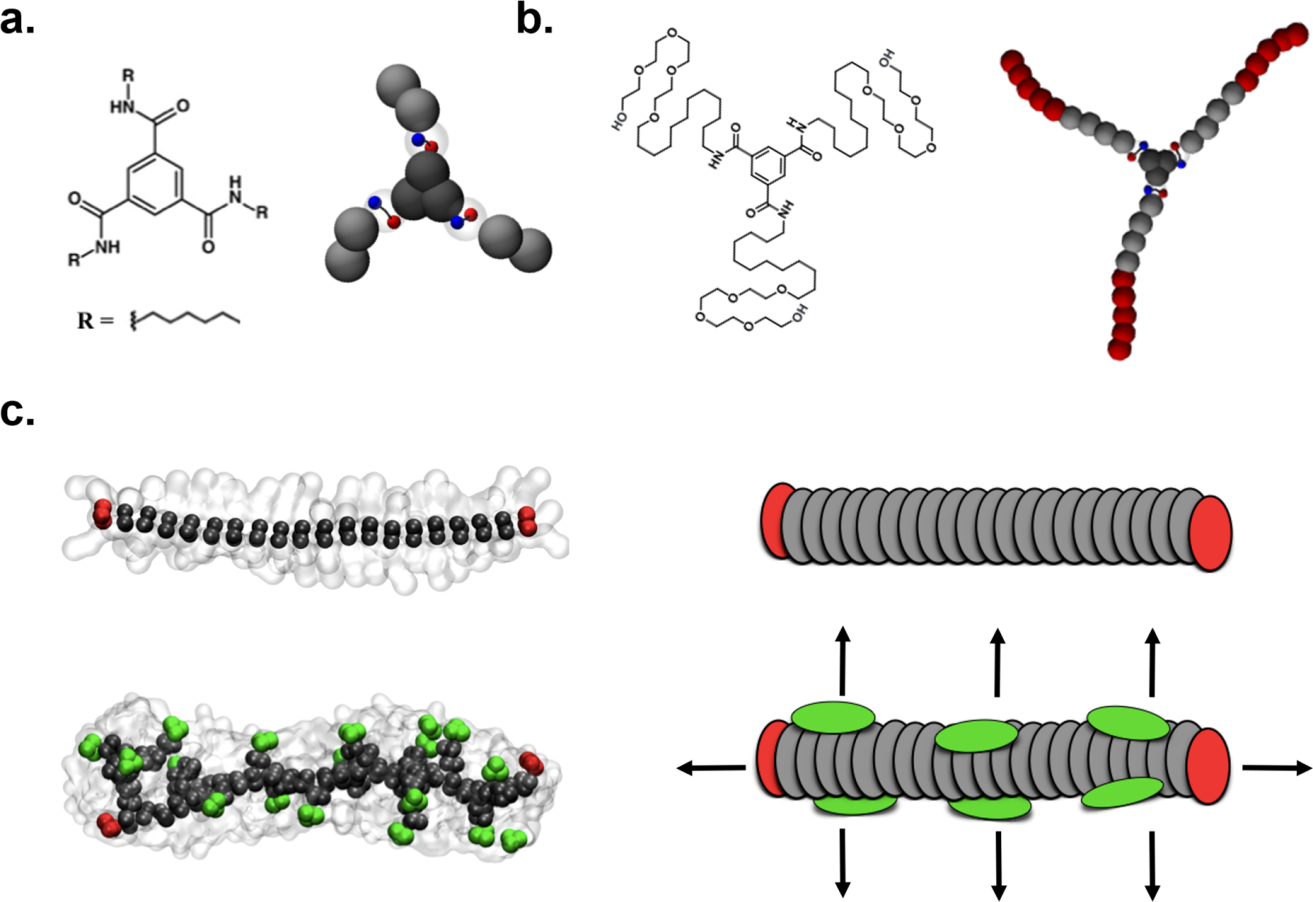
Defects and exchange pathways in BTA supramolecular polymers. (a, b) Chemical structure and CG model of BTA-C6 (a) and BTA-C12-PEG monomers (b) generating supramolecular polymers, respectively, in organic solvent and in water. (c) Equilibrated CG models of octane- (top left) and water-soluble BTA fibers (bottom left);^25^ cartoons show defects in the stacks from which monomers can exchange (green: bulk defects; red: fiber tips).

In general, previous evidence showed that some supramolecular fibers are more prone to exchange monomers from the tips, whereas others exchange from everywhere (tips and side surface).^15^ Molecular models of the water-soluble BTA-C12-PEG supramolecular polymers of Figure 1b show an internal structure rich of defects all along the fiber length, which constitute hot spots for monomer exchange (Figure 1c, bottom: in green).^25^ This explains why these fibers are experimentally observed to exchange predominantly from all along their length.^15^ However, it has been shown that different fibers, such as those formed by the structurally simpler BTA-C6 monomers of Figure 1a designed to self-assemble in organic solvents or by monomers based on other supramolecular motifs, have a more ordered stacked structure, where the two only evident defected points are represented by the fiber tips (where the tip monomers are coordinated with the other monomers in the assembly only on one side).^21,25−27,29^

While intuitively in these assemblies the emergence and the behavior of such defects is encoded into the monomer structure, in the interactions between the monomers, as well as in the interaction between the monomers and the external solution, all these results suggest the intriguing opportunity to learn how to control the exchange pathways in the supramolecular polymers by learning how to control their defects. This fascinating perspective demanded for a more detailed and general-character investigation of the key factors that control the exchange pathways in such complex systems.

Here, we report a minimalistic and rather general CG model that, together with the use of advanced sampling techniques and machine learning, allows us to extract useful information on how to control the formation and the abundance of defects in the supramolecular fibers by controlling the molecular interactions in the system. Combining all our simulation results, we show with a simple kinetic model that the preferred exchange pathway is controlled by the probability of having defects in the self-assembled structures. Thanks to the flexibility and the general character of this minimalistic model, we demonstrate how such probability is directly related to the interactions between the monomers and to the interactions of the monomers with the external solvent. Finally, we provide molecular relevance to the obtained results, showing viable molecular/structural modifications of the monomers that allow controlling the defects and the exchange pathways in the supramolecular polymers.

## Results and Discussion

### Defects and Monomer Exchange in a Reference Family of Supramolecular Polymers

Our investigation starts from a well-studied self-assembling motif, BTA,^30^ that generates supramolecular polymers *via* 3-fold hydrogen bonding and directional stacking of monomer cores (Figure 1). For this first part of the study, we rely on CG models for BTA supramolecular polymers (resolution ∼ 5 Å) that we developed recently.^25^ Based on the MARTINI force field,^31^ our BTA CG models also include rigidly rotating dipoles (Figure 1a,b: ±*q* charges in blue and red), mimicking the directional nature of the inter-monomer H-bonding between the amide groups of the BTAs. We will, later on, generalize our study to obtain broader perspective results.

**Figure 2:**
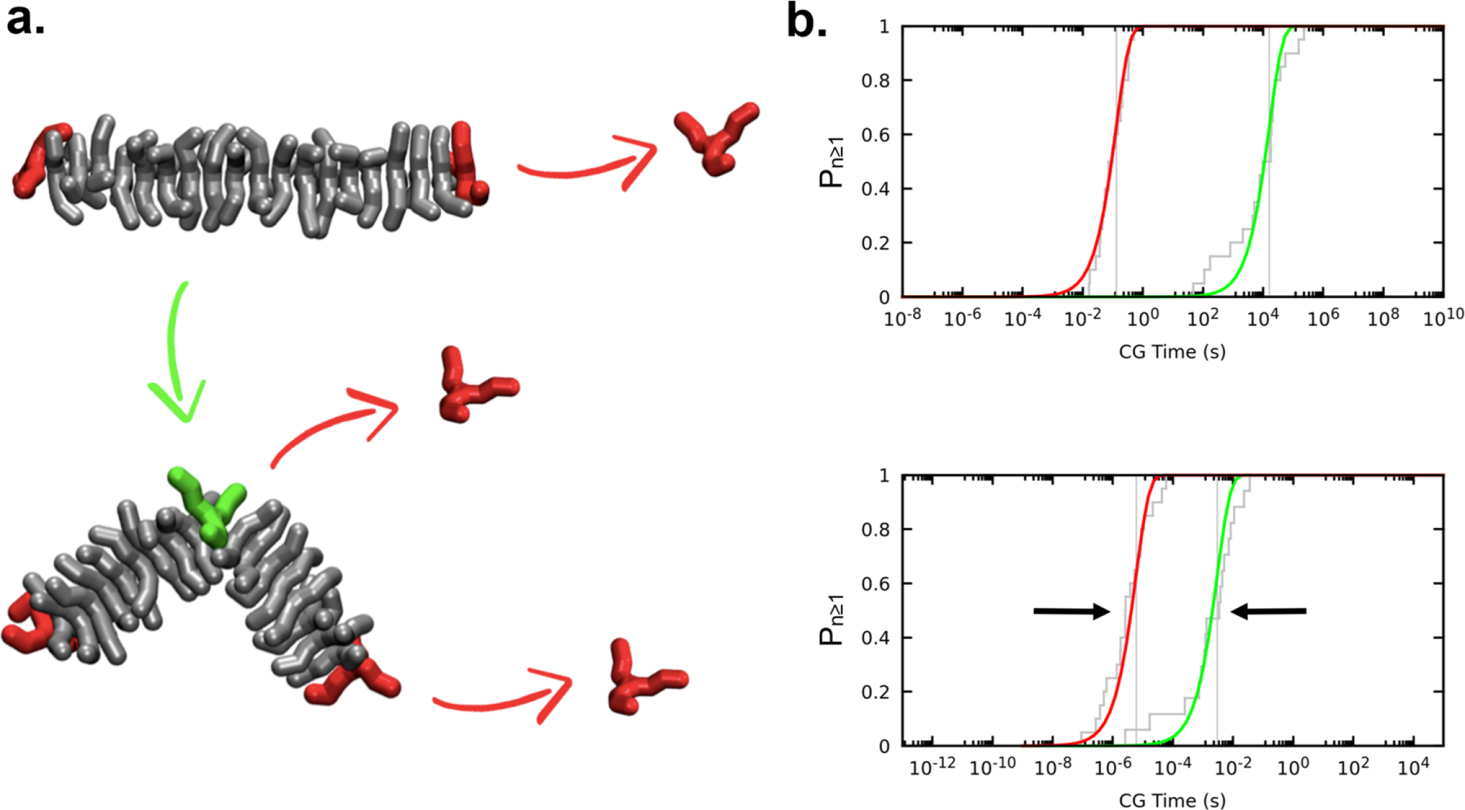
Exchange pathways of BTA supramolecular polymers in organic solvent. (a) The exchange from the fiber backbone is a two-step process, in which, first, a defect (a new tip) is created and, then, the monomer can exchange with the solvent. (b) Cumulative Poisson probability distributions (*P*_*n*≥1_) for the rare events of monomer exchange from the fiber tip (in red) and of generation of a bulk defect along the fiber (in green). These provide an indication of the characteristic time scale for the events (roughly correponding to the midpoint of the Poissonian sigmoidal curves; see Computational Methods). The distributions refer to a BTA-C6 fiber in octane (b, top) and to the same fiber with artificially weakened directional interactions between the monomer cores (b, bottom). In the latter case, the characteristic time scales for the creation of defects and exchange from fiber tips (τ values identified by vertical gray lines) become closer to each other. A similar effect is obtained by artificially increasing the solvophobicity of the monomers (see Figure S1).

The pathways for monomer exchange in BTA assemblies are nontrivial to elucidate. Nonetheless, clear evidence that defects are essential for the exchange of monomers in/out from these fibers has already been provided.^25,29^ When considering a straight and ordered stack of monomers (see Figure 2), we can assume that monomer exchange in these fibers may occur at the fiber tip (*i.e.*, a single step tip-to-solvent event) or at any point of the entire fiber, but only after the creation of a defect (*i.e.*, in a bulk-to-defect plus defect-to-solvent multistep process). In this simple case (BTA-C6 monomers), a bulk defect is equivalent to a new tip. We thus used the coordination of each monomer core with the other cores in the system to distinguish between fiber tips (or defects, coordination 1), perfectly stacked bulk monomers (coordination 2), and monomers in solution (coordination 0). We used our model to investigate the two crucial determinants of the possible exchange pathways: *i.e.*, (i) the exchange of a monomer from the fiber tip (Figure 2a, in red) and (ii) the creation of a defect (equivalent to a new tip) along the fiber backbone (Figure 2a, in green).

Monomer exchange transitions in these assemblies are rare events, occurring on time scales that typically exceed those accessible *via* unbiased molecular dynamics (MD) simulations using such high-resolution molecular models. Thus, similar to what we recently did for water-soluble BTA fibers,^25^ we turned to well-tempered metadynamics (WT-MetaD) simulations.^32^ We ran multiple infrequent WT-MetaD simulations activating monomer exchange transitions (i) and (ii), obtaining transition times distributions (Figure 2b: transparent gray) that fit well with the cumulative Poisson distributions (in red and green) expected for rare events.^25,33^ From the cumulative Poisson distributions, we extracted the characteristic transition times (τ, see the Computational Methods section for details) for the two events (i) and (ii). The results indicate that the creation of a (bulk) defect along the fiber backbone is an extremely rare event compared to the monomer exchange from the fiber tip. The kinetics for the creation of bulk defects is found ∼4 orders of magnitude slower than exchanging monomers from the fiber tip. The associated characteristic transition times obtained from the Poissonian fits of Figure 2b (top) are, respectively, τ ∼ 10^–1^*vs* τ ∼ 10^4^. Such transition time scales τ are obtained from approximated CG models and should be thus considered just as qualitative. However, we can safely compare different processes (τ(i) *vs τ*(ii) - Figure 2b: red *vs* green) as well as fiber variants.^25^ It is now clear that BTA-C6 fibers exchange monomers with the octane solution mainly from the (two) tips. Making an assumption purely based on the estimated transition time scales and on the relative probabilities to observe events (i) and (ii), this remains true for fiber lengths much shorter than ∼2 × 10^5^ monomers (*i.e.*, up to fibers of ∼60–70 μm in length, considering a stacking distance calculated from AA models of ∼3.4 Å).^16,18^ In fact, for such long fibers, the statistical probability of creating defects along the fibers would become non-negligible, and these assemblies would exchange along both pathways.

Recently, we obtained preliminary evidence that the creation of defects (and defect dynamics) may be somehow related to the competition between the directional and the nondirectional interactions between the monomers (*i.e.*, the first one increasing the tendency for these monomers to stack in an ordered way, thus disfavoring the emergence of defects, *vs* the second one favoring a disordered self-assembly).^25,29^ In our case, we can easily test this hypothesis by playing with our CG models in two ways: *e.g.*, (i) by decreasing the charges in the dipoles within the amide CG beads to decrease the directional interactions (*i.e.*, from ±0.8e to ±0.65e) or, *e.g.*, (ii) by changing the beads composing the lateral chains of the monomers, strengthening the nonbond interactions between them, and thus effectively increasing the nondirectional interactions between the monomers (as it would pertain to making the monomers more solvophobic). In (i), the nondirectional interactions between the monomers are kept constant while the directional ones are weakened; in (ii), the directional interactions are kept constant while the nondirectional monomer-monomer interactions become stronger. The results reported in Figure 2b (and in Figure S1 ) clearly demonstrate that, in this way, the rate difference between the two processes decreases. Namely, in the case of decreased dipole charges (Figure 2b, bottom), the difference between the rate (or probability, frequency) of creation of a defect along the fiber (green Poissonian curve: slower event) and the event of monomer exchange from the fiber tip (red curves) is reduced to ∼2.5 orders of magnitude. This means that, compared to the original BTA-C6, reducing the directional interactions between the monomers (the amide dipoles) generates a fiber where the probability of having bulk defects is ∼10^2^/10^3^ times higher relative to the event of monomer exchange from the tip (Figure 2b: red and green curves are closer when the charges in the amide dipoles are reduced to ±0.65e, bottom, compared to the original system, top). A similar effect is obtained also keeping the dipoles as in the standard BTA-C6 model in octane, but increasing the solvophobicity of the BTA side chains (by slightly augmenting the repulsion with the solvent molecules; see Computational Methods section and Figure S1). In such a case, the difference between the time scales for the event of monomer exchange from the tip *vs* the event of creation of a new defect along the fiber is again reduced to ∼10^2^/10^3^ (see Figure S1). In both cases, such fibers would exchange mainly from the tips only up to fiber lengths of ∼70–700 nm in length (composed of ∼2 × 10^2^/10^3^ monomers), while, for longer fibers, the probability to have defects emerging along the fibers from which monomers can exchange would become statistically predominant.

These results support the fact that the competition (ratio) of directional *vs* nondirectional interactions between the monomers is important in dictating the preferential pathway along which these fibers exchange monomers with the surrounding. However, this CG model provides limited flexibility for exploring further the exchange behaviors of such supramolecular polymers. For example, a further increase of the solvophobicity of the side chains of the monomers produces 3D aggregates (nanoparticles) instead of fibers with a higher number of bulk defects. This is due to an intrinsic limit of this BTA-C6 model, which is too specific and provides limited space for customizations and for modulating the monomer properties/features. In fact, as well exemplified by the water-soluble BTA monomer of Figure 1b, such fibers require a solvophilic shield in order to be stabilized in the solvent (*e.g.*, such as the PEG chain ends in this BTA variant).

### Generalized Minimalistic Model

**Figure 3:**
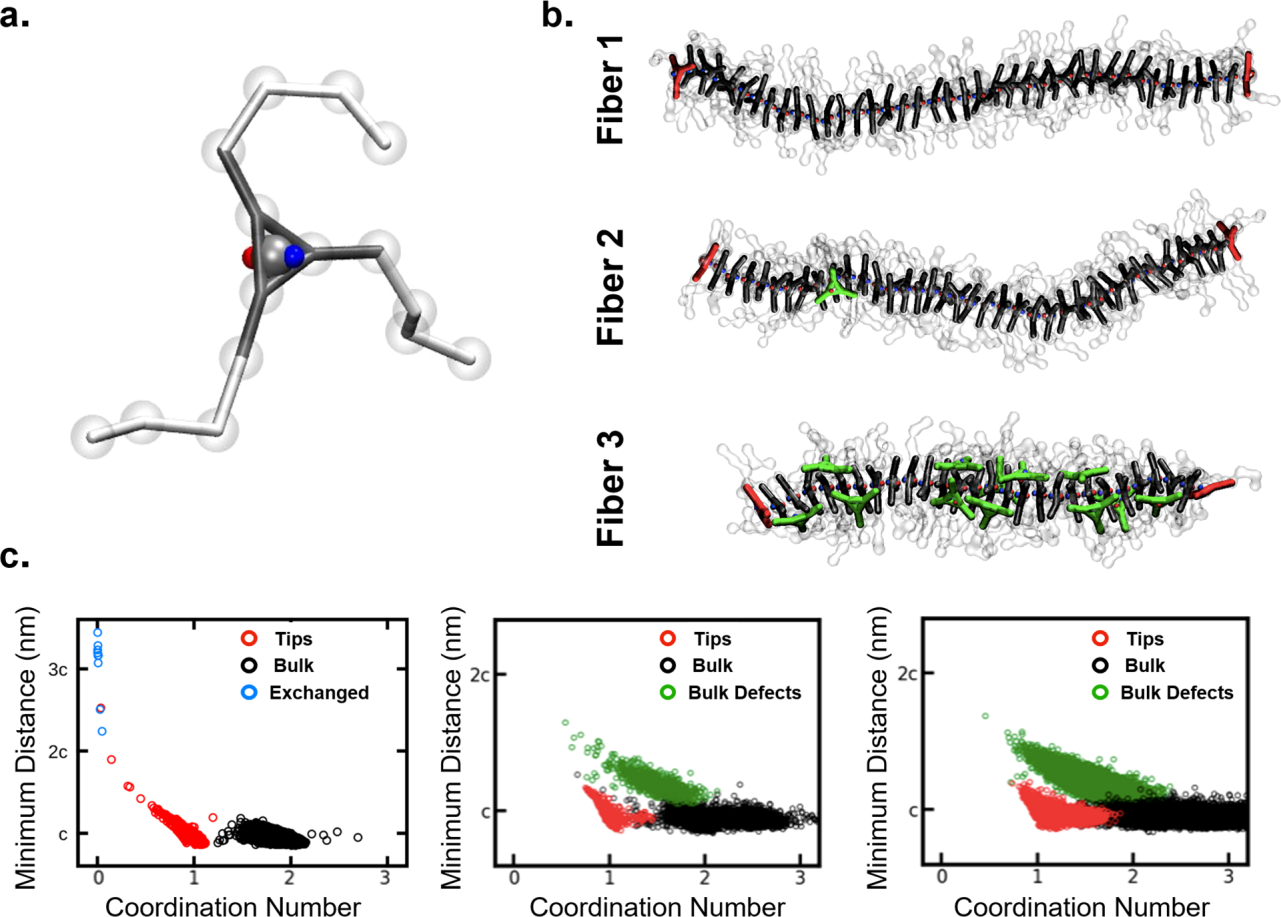
Defects in coarse-grained models of supramolecular polymers. (a) Minimalistic CG model for the 3-arm self-assembling monomers studied herein. A dipole (blue and red) in the central bead provides directional interaction between the cores. (b) Three fiber variants composed of 40 monomers with different directional *vs* nondirectional interactions balance: Fiber **1** (completely solvophilic monomers self-assemble only due to directional interactions), Fiber **2** (interior of the monomers slightly more solvophobic than in Fiber **1**), and Fiber **3** (monomers interior more solvophobic than in Fiber **2**). (c) Supervised clustering analysis of the fibers, identifying different structural motifs: bulk monomers in black, fiber tips in red, bulk defects in green, and exchanged monomers in blue. Data are reported for fibers **1**, **2**, and **3** (left to right).

To generalize our study, we developed a minimalistic (coarser) CG model, allowing us to simulate these systems on a higher-scale. Such a model loses chemical accuracy, while, at the same time, this is representative of a wider class of supramolecular polymers: *e.g.*, of self-assembled fibers composed of monomers having three side arms (see Figure 3a). In this CG model, the monomers interact directionally *via* a dipole inserted in the central bead of the monomer structure (blue and red). Such a central dipole, replacing the three original dipoles present in the amides CG beads of BTA-C6 model, aims at representing the directional interactions between the monomers in a more abstract way. Nondirectional interactions are present between all CG beads composing the monomer (core and side arms) in the typical form of a Lennard-Jones potential.

We compared three fiber variants, each composed of 40 identical monomers, which differ only in the hydrophobicity of the inner part of their monomers. In detail, the three terminal beads of each side chain (connected by white bonds in Figure 3a) are always kept solvophilic, while two internal ones (connected by gray bonds) are composed of more/less solvophobic CG beads, depending on the fiber/monomer variants. In monomer **1**, all CG beads in the side arms are identical to those of the solvent (for simplicity, we used C1 MARTINI beads). In this case, the monomer structure is completely solvophilic since the explicit solvent in the simulation box is also composed of C1 beads, and the monomers self-assemble only due to the directional interactions between the central dipoles. Monomers **2** are identical to monomers **1**, except for the first two inner beads in each side arm, which are more solvophobic (the C1 beads are replaced with C5 MARTINI beads in this case, less affine to the solvent molecules). The solvophobicity of the internal part of the monomers is then increased further in Fiber **3**, the inner CG beads of monomers **3** being modeled using N0 beads in this case (which makes the internal part of monomers **3** the least affine for solvent). The central dipole was optimized in order to reproduce with the CG model of monomers **3** the dimerization free energy of the water-soluble BTA-C12-PEG monomers of Figure 1b in water (∼10 kcal mol^–1^)^19,25^ and was kept constant in all cases. In terms of assembled fibers, Fiber **1** is thus similar to BTA-C6 fibers in (good) octane solvent, while Fiber **3** (composed of monomers having amphiphilic arms: a solvophobic interior and a solvophilic surface) is reminiscent of BTA-C12-PEG supramolecular polymers in water (Figure 1b).^16,18,19,25^ Complete details of the CG models are provided in the Computational Methods section and in the Supporting Information.

Starting from an initially perfect stack of extended monomers, we simulated the three fiber models for 30 μs of unbiased CG-MD simulation, obtaining quite different equilibrium structures (see Figure 3b). Fiber **1** appears as straight and linear, preserving the initial monomer stacking and the internal order of the cores. Conversely, the equilibrium structure of Fiber **3** presents numerous defects all along its length—green monomers which are still part of the assembly, but not stacked in an ordered way in the fiber backbone. This is reminiscent of what happens in finer (higher-resolution) CG molecular models of BTA-C12-PEG fibers in water.^25,29^ Fiber **2** is somewhat intermediate: a few defects appear and disappear along the fiber backbone during the CG-MD run. Interestingly, in this case, defects are not always present (persistent in time) but they are created and re-adsorbed in a dynamic way in equilibrium conditions (Figure 3b). In general, this minimalistic CG model allows us to qualitatively retrieve differences in global structural features, such as, *e.g.*, the internal order, presence of defects, *etc.*, seen in different BTA supramolecular polymer variants by tuning only the relative strength of nondirectional interactions *vs* directional interactions, thus, by playing only with one parameter, *i.e.*, the solvophobicity of the inner monomer beads (given that the dipoles are kept constant in this comparison).

To quantify and compare the defectiveness of the different fibers, as a first step, we used a supervised clustering analysis of the equilibrium CG-MD trajectories, exploiting four relevant structural collective variables (calculated for each monomer) and applying a spectral clustering-based algorithm^34^ to distinguish the different structural motifs present in the fiber structures (see Computational Methods for further details). The results of this clustering analysis are reported in Figure 3c, where the clusters are projected on two variables: the coordination number (here, 2 indicates perfectly ordered stacking, as each core in a perfect stack has exactly two closest neighbors) and the minimum distance from the other monomer cores (*c* is the stacking distance between two parallel neighbor cores in a perfect stack).

Using spectral clustering to divide the monomers of Fiber **1** into three main clusters, we can recognize in them different physical states (see Figure 3c). In black, monomers belonging to the bulk of the fiber are perfectly stacked/ordered (coordination number 2 and minimum distance *c*). Monomers at the fiber tips are colored in red (coordination number 1 and minimum distance *c* - monomers stacked only by one end). The blue cluster identifies those monomers that spontaneously leave (exchange out from) the fiber during the CG-MD run, thus having minimum distance > 2*c*. Interestingly, the enhanced sampling granted by this minimalistic CG model allows us to observe monomer exchange events even during an unbiased CG-MD simulation. The CG-MD trajectory shows that Fiber **1**, representative of a supramolecular polymer where the monomers are well solvated and self-assemble only due to directional interactions, predominantly exchanges monomers out from the fiber tips, or, more rarely, from fibers breakage that might occur during the dynamics (see Movie 1). Noteworthy, in this case, we do not observe the formation of any stacking defect or disordered domain along the fiber.

The same analysis for Fiber **2** and Fiber **3** instead reveals different clusters: bulk ordered/stacked monomers (in black), monomers at the fiber tips (red), and bulk defects along the fiber (in green). Bulk defects (in green) are present in both cases, but the density of defected green points is rather different in the two fibers. We observe that the average number of bulk defects present in the CG-MD equilibrated simulations is slightly less than 1 for Fiber **2** (∼0.8) and ∼12 for Fiber **3**. Noteworthy, in Fiber **2**, the average number of defects in the stack is <1, which means that defects do not have a persistent nature. Namely, in this fiber, defects are not always present but they dynamically form and re-adsorb along the fiber. On the other hand, Fiber **3** has a much higher intrinsic number of persistent defects distributed all along the fiber. In fibers **2** and **3**, the blue cluster is not present, as the increased solvophobicity of the monomers (producing an augmented interaction between the monomers) does not allow observing spontaneous monomer exchange out from the fiber during an unbiased CG-MD simulation. This again fits well with recent enhanced sampling simulations showing that monomer exchange events slow down, and the fibers become overall less dynamic, as the solvophobic interaction between the monomers is increased in the assembly.^25^

**Figure 4:**
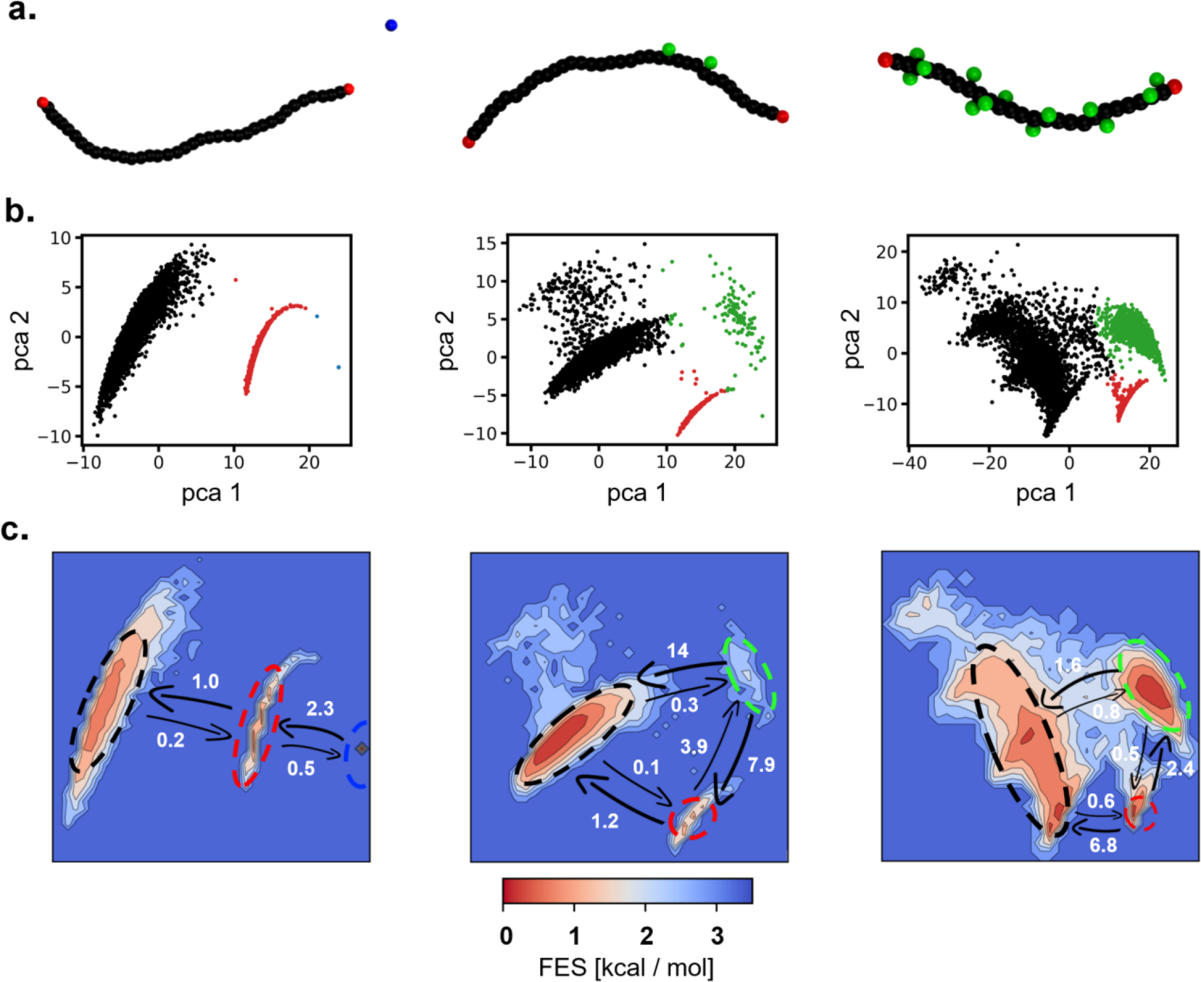
Unsupervised machine learning of defects and of defect dynamics. (a) Minimalistic representation of the three fiber models, showing only the center for each monomer in the fibers. The monomer centers are colored according to the cluster these belong to (black: ordered bulk of the fiber; red: fiber tip; green: bulk defects; blue: exchanged monomers). (b) First two principal components (PCA 1 and PCA 2) obtained from the dimensionality reduction of the SOAP analysis of the equilibrium CG-MD trajectories of the three fibers (PCA models trained separately for each fiber). Scatter plots are colored according to the main macro-cluster obtained from the PAMM analysis. (c) Low dimensional free energy surfaces of the three fibers, computed from the monomer states distributions of panel (b), showing the kinetic analysis of the relative transition rates between the macro-clusters. Each macro-cluster depicted in the FES as a dashed ellipsoid, roughly representing the area of that cluster. The arrows represent the transition rates for the interconversion of macro-clusters along CG-MD trajectories; all numbers reported are in 10^6^ s^–1^ units, which are left out for clarity (CG transition rates, having a comparative value).

To enrich our analysis through a general approach, we also used a more advanced and abstract unsupervised machine learning approach to compare the fibers.^29^ In detail, we analyzed the equilibrium CG-MD trajectories of the three fiber CG models with a combination of high-dimensional molecular descriptors (*i.e.*, the smooth overlap of atomic positions, SOAP, vectors)^35^ and an unsupervised density-based clustering technique, *i.e.*, the probabilistic analysis of molecular motif, PAMM^36^ (see Computational Methods for details). This high-dimensional analysis is more agnostic, in that it does not require one to select in advance critical variables for the identification of the clusters, nor the clusters number. This method allows us to obtain at once the micro- and macro-clusters in the system, representative of the dominant structural states for the monomers (and their surroundings) in the fibers, their similarity, and dynamic interconversion. We come out with a throughout structural and dynamic characterization of these fibers useful to compare between them.^36^ The results are summarized in Figure 4, while the complete analysis for the three fibers is reported in Figures S2–S4.

First, considering all monomer cores in the CG-MD trajectories of the three fibers as unique data set, this approach allows us to subdivide the monomers along the CG-MD trajectories into clusters that are (qualitatively) comparable between the three systems (see Computational Methods for details).^36^ Namely, this allows us to attribute to the different monomer states in Fibers **1**, **2**, and **3** the colors to the clusters in a transferable way (Figure 4a,b: black, red, green and blue clusters). The macro-clusters obtained *via* this bottom-up unsupervised analysis identify structural features and differences in these fibers that are consistent with those of Figure 3c: bulk monomers, fiber tips, defects, and, only for Fiber **1**, monomers exchanged with the solution.

From the data sets collected from the equilibrium CG-MD trajectory of each fiber, it was also possible to reconstruct the free energy surface (FES) of the internal structure of these fibers, of the molecular motifs present therein, and also to obtain an insight into their dynamic interconversion, *i.e.*, on the internal structural dynamics of the fibers. Figure 4c shows the FES of the three fibers and the relative free energy of the macro-clusters, whose regions are depicted as a dashed ellipsoid, present in the fibers (represented in the space of the first two principal components, PCA 1 and PCA 2).

From this SOAP-PAMM analysis, it is also possible to monitor the monomers that dynamically change cluster during the CG-MD trajectory and to obtain interesting information on how comparably faster/slower the monomers exchange between the clusters in the various fibers, *i.e.*, comparing the supramolecular dynamics of the fibers. The data of Figure 4c indicate that, in these fibers, there is a continuous interconversion between these monomer states. While the transition rates between the clusters of Figure 4c (black arrows) are estimated from approximated CG models, these still maintain a qualitative value and are useful to compare the transitions between the states within the same fiber, and to qualitatively compare the structural dynamics of the three fibers.^36^ Particularly interesting is the green minimum appearing in Fiber **3** (Figure 4c, right). Whereas, in this fiber, bulk defects constitute a persistent and minimum energy state, in Fiber **2**, this state is ∼2 kcal/mol higher in free energy compared to the global minimum (black cluster: ordered monomers in the fiber bulk). This is the reason why bulk defects form only intermittently in Fiber **2** and do exist in this fiber (differently from Fiber **3**) in a purely statistical fashion (see also Movie 2). In particular, in Fiber **2** the ratio between the rates of annihilation/creation of bulk defects (into/from ordered bulk domain) is ∼40 (green-to-black ∼ 14 μs^–1^*vs* black-to-green ∼ 0.3 μs^–1^). Conversely, in Fiber **3**, the two rates are comparable (their ratio being reduced to ∼2), as, in this case, black and green states exist as “persistent” states, *i.e.*, as local energy minima separated by a free energy barrier of ∼2.5–3 kcal/mol (see also Movie 3). As in Figure 3c, also in this unsupervised clustering analysis, the green cluster is not present in Fiber **1**.

These results are interesting in light of the fact that defects work as hot spots for monomers exchange in/out from and within such supramolecular fibers.^11,25,29^ For example, they indicate that a supramolecular polymer where the monomers are self-assembled purely due to directional interactions may break, or it may exchange monomers out from the fiber tips, but it does not create bulk defects along the stack, as the driving force for such an event—*i.e.*, nondirectional interactions—is absent (see also Movie 1). Also, these data suggest that, in general, a supramolecular polymer full of defects is likely to exchange monomers from all along its length/surface, similar to what has been already seen for BTA-C12-PEG fibers in water.^15,16,23,25,37^

### Determinants of the Exchange Pathway

**Figure 5:**
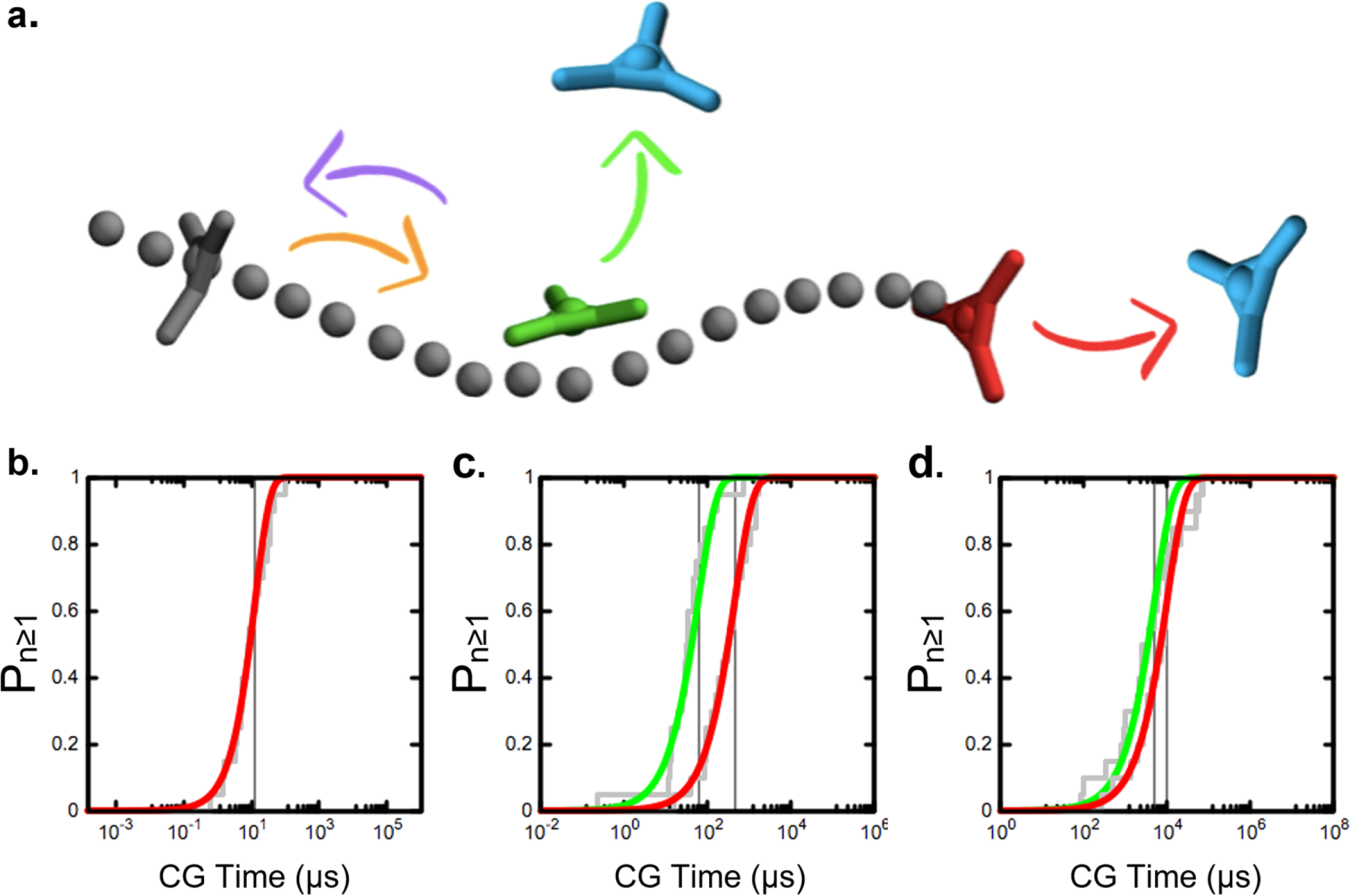
Monomer exchange pathways in a supramolecular polymer. (a) Scheme of rare events in the exchange pathways: exchange with the solution from the tip (red arrow) or from a bulk defect (green). The events of creation (orange) and annihilation (purple) of a bulk defect have a statistical nature. (b–d) Cumulative Poisson distribution fits for the events of exchange with the solution, respectively, for Fiber **1** (exchange from the tip in red), Fiber **2**, and Fiber **3** (exchange from the tip in red or from a bulk defect in green). The characteristic transition time scales (τ) related to each distribution are identified by vertical gray lines.

Given that the abundance of defects in these fibers appears to be connected to the degree of solvophobicity of their monomers (*i.e.*, to the effective relative magnitude of the nondirectional interactions between the monomers *vs* the directional ones), it is desirable to identify an indicator capable of distinguishing from where a given fiber is going to preferentially exchange monomers. Namely, this would allow us to infer/predict the exchange pathways in the assembly. In general, the possible pathways for monomer exchange out from a supramolecular polymer are schematized in Figure 5a: red arrow for an exchange from the tip, and green arrow for an exchange from a bulk defect. We defined a dimensionless parameter α in this way
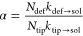
1where the number of fiber tips *N*_tip_ is 2 by definition, *N*_def_ is the average number of bulk defects at the equilibrium, *k*_def→sol_ is the rate of monomer exchange with the solution from a defect, and *k*_tip→sol_ is the rate of exchange with the solution from a tip. The average number of defects in the two fibers has been calculated *via* block average (block size of 5 μs) in two ways: using the supervised classification of Figure 3c, and using the unsupervised SOAP-PAMM classification of Figure 4. The two methods provided consistent results: an average number of defects slightly less than 1 for Fiber **2** and ∼12 for Fiber **3**. On the other hand, the exchange with the solution has to be activated in the simulations, since it is a rare event, very difficult to observe with satisfactory statistics *via* unbiased CG-MD (especially in Fibers **2** and **3**, where monomer exchange out from the fiber becomes slower due to the addition of nondirectional solvophobic interactions). As recently done for other supramolecular polymers,^21,25−27^ we turned to infrequent WT-MetaD simulations to activate and to study monomer exchange out from the fibers.^25,38^ In particular, running multiple infrequent WT-MetaD simulations activating monomer exchange out from the fiber tips or out from green defects allowed us to reconstruct the transition probability curves. The statistics for the events of monomer exchange from the fiber tip and from bulk defects are reported in Figure 5b–d. These fit well with the Poissonian statistics expected for rare events (Figure 5b–d) while, as in Figure 2, from these, it is possible to estimate the characteristic transition time scales (τ values identified by the vertical lines) expected for the unbiased exchange transitions (the rates related to these exchange events calculated as τ^–1^).^25,33,38^ It is worth noting that, for Fiber **1**, compatibly with the accuracy that can be expected from such a biased method, the infrequent WT-MetaD simulations provide an estimated characteristic time scale for the exchange of one monomer from the fiber tip to the solution in the order of microseconds (Figure 5b). This is consistent with the characteristic time scale estimated for the same transition in the same fiber *via* unbiased CG-MD (Figure 4c, left: red-to-blue transitions occurring with a rate of ∼0.5 μs^–1^, and thus on a characteristic time scale of ∼2 μs), which proves the robustness of the WT-MetaD setup.

Combining the average number of defects with the estimated exchange rates, we get an α of ∼4 for Fiber **2**. This value is higher than 1, thus indicating that, in this fiber, exchanging monomers from bulk defects (*i.e.*, from all along the fiber surface) is already more likely than exchanging monomers from the tips, even though the two processes are in close competition. Clearly, for a much longer fiber, the exchange from the tips will become more and more unlikely, while exchanging from defects that statistically appear along the fiber will prevail as the most likely exchange pathway. Conversely, in shorter fibers **2**, exchanging from the fiber tips would be predominant. For Fiber **3**, we obtain an α of ∼12. This value is even higher than that of Fiber **2**, indicating that, in this fiber, the most favorable pathway for monomer exchange is from the defects along the fiber.

Equation 1 thus allows us to determine the statistically most favorable exchange pathway starting from a few parameters obtained from (unbiased and biased) molecular simulations of a given fiber. However, it is worth noting that we can use eq 1 only if we already know, or we can estimate with good confidence, the average number of defects in a certain fiber (*i.e.*, if the fiber model is well sampled at the equilibrium). But what regulates the number of defects present into a fiber in equilibrium conditions? At equilibrium, assuming that the exchange in/out from the fiber is much slower than the exchange within the fiber (which is the case for both Fibers **2** and **3**), the following equation should hold
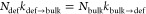
2where *k*_bulk→def_ and *k*_def→bulk_ are, respectively, the rate of creation and annihilation of a bulk defect. Given that *N*_bulk_ = *N*_tot_ – 2 – *N*_def_—where *N*_bulk_ is the number of monomers in the ordered bulk of the fiber (in black), *N*_tot_ is the total number of monomers in the fiber model, 2 is the fiber tips, and *N*_def_ is the number of bulk defects (green)—we can simply estimate the average number of defects from the rates of their creation and annihilation as in eq 3:
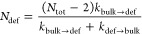
3

The rates for defects creation/annihilation (*k*_bulk→def_ and *k*_def→bulk_) can be obtained from the SOAP-PAMM analysis of Figure 4c (black-to-green and green-to-black transitions). Inserting them inside eq 3, we find an average number of defects (∼0.9 for Fiber **2** and ∼12.9 for Fiber **3**) which is consistent with what was obtained before from our clustering analyses. This is a proof that our simulations are at equilibrium. Therefore, we can safely combine eqs 1 and 3 writing a single general eq 4:
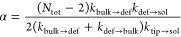
4

Equation 4 has the advantage of containing only rate constants and is thus generally applicable to a variety of supramolecular systems. In the cases studied herein, the creation/annihilation of defects occurs at the equilibrium, and thus the corresponding rates can be estimated directly from unbiased MD simulations. However, for cases in which the slow dynamics of the assembly prevents the observation of such transition events during a classical MD run, the most favorable exchange pathway can be still deduced by using biased infrequent WT-MetaD simulations to infer the transition rates for both key steps: (i) creation and annihilation of defects and (ii) monomer exchange with the solution from tips and defects.

### Toward Controlling Defects and Exchange Pathways in Supramolecular Polymers

Our analysis shows that, under equilibrium conditions, the average number of defects in a fiber is directly connected to their creation and annihilation rates (probabilities), *k*_bulk→def_ and *k*_def→bulk_, respectively. As demonstrated by the comparison between Fibers **1**, **2** and **3**, this is clearly related to the balance between directional and nondirectional interactions between the building blocks, which dictates the average features for the fibers in terms of average number of defects and thus of dominant exchange pathways.

**Figure 6:**
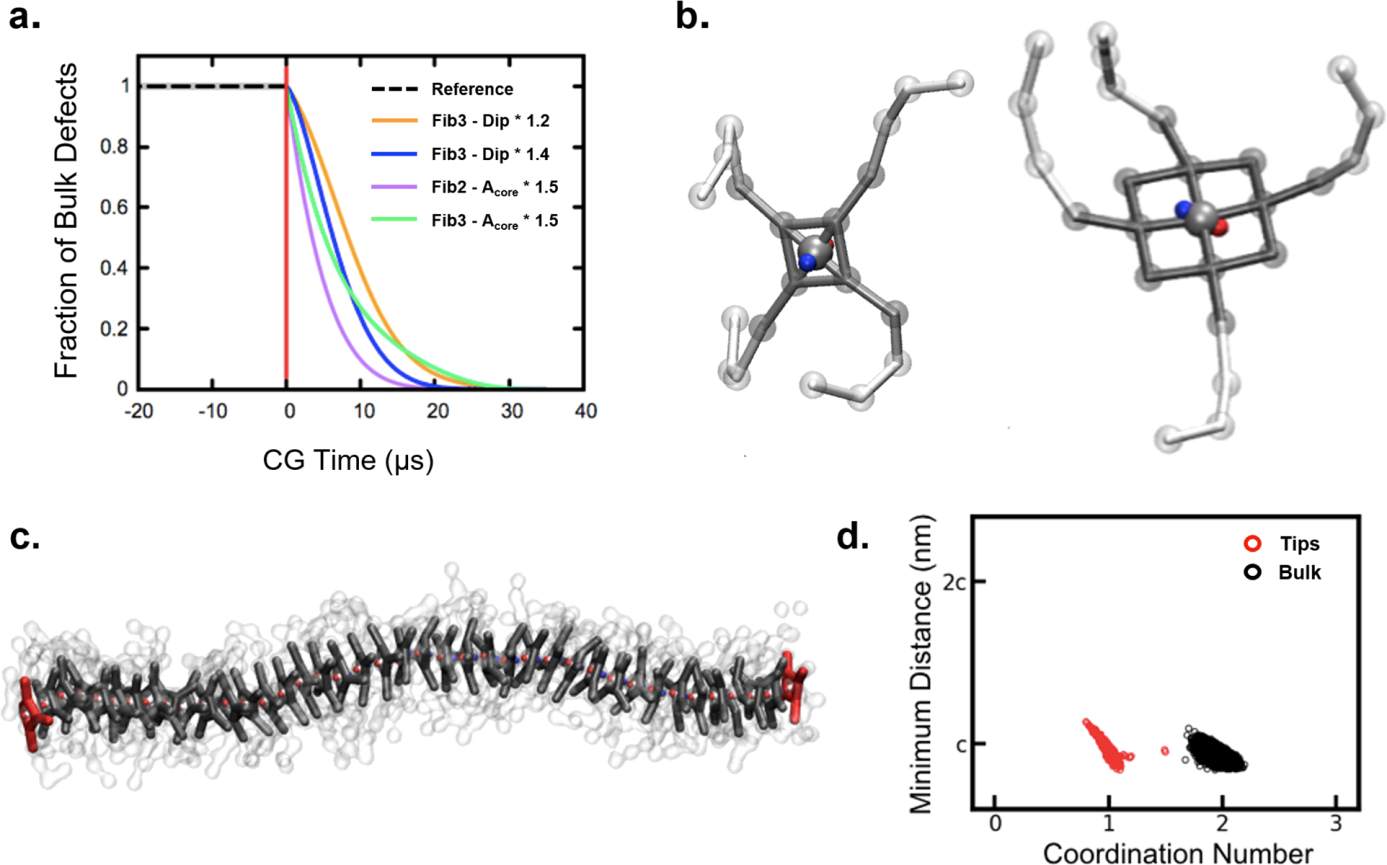
Controlling the number of defects by changing monomer-monomer interactions or monomer shape factors. (a) Decreasing the number of defects by increasing directional interactions. On the left of the red vertical line: normalized fraction of defects in the fibers at the equilibrium (our reference). On the right of the red vertical line: change in the normalized fraction of defects as a function of simulation time obtained by (i) increasing the charges in the central dipole (Dip) of Fiber **3** by a factor ∼1.2 (orange) or ∼1.4 (blue), or (ii) by increasing by a factor 1.5 the monomer core area (*A*_core_) in both defected fibers (Fiber **2** in purple and Fiber **3** in green). All the curves have been smoothed with Bézier curves. (b) Minimalistic CG models for monomer with four-arm cores. Left: A monomer with the same core area as BTA (but having square instead of triangular shape) and the same amphiphilic arms of Fiber **3**. Right: a monomer with the same core area as a porphyrin.^21^ (c) Fiber composed of 40 four-arm monomers having a porphyrin core and the same amphiphilic arms of Fiber **3**, the same as in (c, right). (d) Equilibrium macro-clusters for monomer states in the fiber of (c): fiber tips in red and bulk monomers in black. Bulk defects are completely absent.

Therefore, by modulating the nondirectional *vs* directional interactions balance, one should find that the number of defects also changes going toward a new equilibrium: the relatively stronger will be the directional interactions between the cores, the more straight and flawless the fiber will become. As a proof of concept, we considered the equilibrated models of Fibers **2** and **3**, and we changed the strength of directional interactions over nondirectional ones. This can be done, for example, (i) by increasing the dipole charges or (ii) by increasing the size of the (flat) monomers core, enhancing the cores’ tendency to stack. Figure 6a shows that, starting from the equilibrium (defected) structure of Fiber **3**, the defects population disappears for these cases during CG-MD simulations, providing equilibrium fiber structures without any bulk defect. The orange and the blue curves represent the number of defects (normalized *vs* intrinsic number of defects of Fiber **3** at the equilibrium, set to 1) while increasing the dipole charges from the original 1.45e to 1.7e or to 2e. On the other hand, the green and the purple curves refer to the case in which the triangular monomer core area was increased by a factor 1.5. In all these cases, the number of defects drops to zero, and after 30–40 μs of CG-MD, all fibers appear as almost completely straight and defect free. This shows the distinct connection between the interactions balance and the amount of defects along the fibers, which, for all that was said above, controls what exchange pathway is the most favorable in a given fiber.

We also explored the effect of changing the number of the monomer arms (while preserving their same amphiphilic nature as in Fiber **3**). Since the side arms end with solvophilic beads, we expected an improved screening effect against the formation of defects along the fiber by adding one more arm. We built two versions of a four-side-arm monomer with a square-shaped core: one having the same core area of the original (triangular cored) monomer model and the four arms grafted onto the squared core vertexes, and a second one, with the same core area of a porphyrin (Figure 6b). In particular, the latter model is reminiscent of self-assembling monomers having a porphyrin core generating straight and defect-free supramolecular polymers in organic solvent.^21^ While these changes represent only a small number of all possible monomer customizations, they serve here as proof-of-concept validations, showing viable ways (*e.g.*, reminiscent of realistic molecular customizations)^21,22^ to control the abundance of defects and the exchange pathways in the supramolecular polymers. In both cases, the self-assembly of monomers results into fibers without bulk defects as shown in Figure 6c,d. This demonstrates how molecular factors such as, *e.g.*, the solvophobicity and shape factors of the monomers concur in controlling the competition between nondirectional and directional interactions in the system, dictating not only the structural features of the fiber but also its internal dynamics and, ultimately, the exchange pathways.

## Conclusions

Here, we have developed a minimalistic model for supramolecular polymers that, starting from realistic molecular systems, allows us to explore the effects of general features in the monomers on the structure, dynamics, and exchange pathways of the supramolecular polymers. We combined multiscale modeling, classical and advanced simulations, and unsupervised machine learning to obtain a thorough characterization of the internal structure and dynamics of different types of supramolecular fibers. Our data demonstrate the intimate connection between the defects that are present or that may form along the fibers and the most favorable pathways for monomer exchange in the assembly. By controlling defects, it is possible to control the exchange of monomers in the fibers, in terms of both exchange kinetics/frequency and pathways. The formation of defects can be then controlled by controlling the competition between directional *vs* nondirectional interactions between the self-assembling monomers. The general nature of our CG models allows to obtain general knowledge on key factors that control the dynamic behavior of such complex systems. At the same time, it also allows us to provide chemical relevance to the obtained results, showing for example how changing the aspect ratio of the monomer cores to increase/reduce the directionality of the self-assembly may allow reducing/augmenting the statistical chance to form defects along the fiber, moving the most favorable pathway for monomer exchange from the fiber tips to the (defected) fiber backbone/surface. We come out with general concepts, which increase our comprehension of how it is possible, in principle, to rationally design supramolecular polymers that exchange the constitutive building blocks with the external environment according to programmable pathways.

## Computational Methods

### Creation and Parametrization of the CG Models

The starting fine CG model for the BTA-C6 supramolecular polymer in octane solvent of Figure 2a is taken from our previous works.^19,25^ This is based on the MARTINI coarse-grained force field,^31^ which guarantees a good transferability while globally preserving the thermodynamic properties of the mapped species. In this model, the aromatic core is composed of three SC5 beads, onto which three CG beads representing the amides are connected, which contain three rigidly rotating dipoles (mimicking the amide–amide hydrogen bonding).^19,25^ The core of the BTA-C6 monomer model is connected to three short hydrophobic (and thus solvophilic, as the solvent is modeled *via* explicit octane molecules) side chains, *i.e.*, two SC1 MARTINI beads, corresponding to 6 carbon atoms (see Figure 2a). When studying the effect of the variation of the directional *vs* nondirectional interactions, we modified this model by either decreasing the value of the charges in the amide CG beads (from ±0.8e to ±0.65e - weakening the directional interactions between the monomers) or by changing the CG beads composing the hydrophobic tails (from SC1 to SC5 MARTINI beads), making them less solvophilic (*i.e.*, more solvophobic), considering that the explicit solvent is octane, modeled with two C1 beads (SC1 and C1 beads have the same solvophobicity, while C5 interacts less favorably with the C1 solvent and is thus more solvophobic).

The minimalistic CG model for BTA that is used in the second part of this study has been developed, for simplicity, using as a basis the BTA-C6 MARTINI-based model described above. The global CG structure of the monomer model was substantially preserved: the flat monomer core is composed of three CG beads, shaped as an equilateral triangle (as in the BTA-C6 model) onto which three arms are grafted, each composed of four beads: initially, four identical solvophilic C1 beads, considering that the external explicit solvent is also composed of C1 beads. The directional interactions between the monomer cores have been modeled by adding one central (P5) bead containing a dipole in which the two partial charges (+*q* and −*q*) were kept at a fixed distance. Thus, this minimalistic model replaces the 3 amide beads (and the 3 amide dipoles) with one single dipole placed at the center of the core, optimized in order to have a comparable effect on the monomer-monomer interaction. In particular, in the minimalistic CG model, the dipole charge value (± 1.45e) was chosen in order to obtain for monomers **3** the same dimerization free energy of BTA-C12-PEG in water (∼10 kcal mol^–1^),^19,25^ and then it was kept constant for the three variants of the model (Fibers **1**, **2** and **3**). This allowed us, for simplicity, to use the MARTINI force field nonbond interactions to tune the Lennard-Jones epsilon, and thus the interactions between the beads that compose the core (monomer-monomer and monomer-solvent). In the comparisons of Figures 3–5, the internal monomer beads (*i.e.*, the first 2 arms CG beads) have been then changed from C1, in Fiber **1**, to C5 and N0, in Fibers **2** and **3**, respectively, thus increasing the inner solvophobicity of the monomers. The complete topology of our generalized CG model (.itp files, GROMACS format) is provided in the Supporting Information.

In the final analysis of Figure 6, the geometry of the central core of the generalized CG model has been modified in size, by increasing the area of the core triangle by a factor 1.5 (*i.e.*, larger triangular core). This allowed us to investigate the effect of the core planarity (ratio between core area and core width) on the directional interactions between the monomers and on the formation of defects within the fiber. In the same spirit, to study the effects of increasing the number of side chains, we constructed an equivalent model with square-shaped cores (see Figure 6b) composed by 4 beads (same beads of the triangular core). The square side was chosen in order to obtain the same area of the first reference triangle-shaped core. Then we constructed also a square core composed by 9 beads to study the effect of varying the planarity in the case of a square core.

### MD Simulation Parameters

All MD simulations were carried out with the GROMACS 5.1.2 software^39^ in *NPT* conditions (constant *N*, number of particles; *P*, pressure; *T*, temperature during the run). We used a 20 fs time step, a straight cutoff (1.1 nm) combined with potential modifiers and the Verlet neighbor list scheme,^40^ the V-rescale thermostat,^41^ and the Berendsen barostat.^42^ In all simulations, the temperature was kept at 300 K with a coupling constant of 1.0 ps, and the pressure was maintained at 1 atm with a coupling constant of 2 ps. Production runs with the three minimalistic model variants had a total duration of 30 μs. The first 10 μs (equilibration time) was excluded from the analysis.

All metadynamics simulations were conducted using the PLUMED 2 plugin.^43^ Our approach builds on the work of Tiwary and Parrinello, demonstrating that the kinetics of an event (*e.g.*, defect creation, or monomer exchange out from a defect) can be efficiently calculated from the transition time obtained from biased infrequent WT-MetaD simulations.^33,38^ The approach is valid provided that the collective variables (CVs) along which the bias is applied are opportunely chosen and that the bias is not deposited on the transition barrier during the (infrequent) WT-MetaD runs.^38^ Moreover, the reliability of the obtained kinetics statistics can be systematically verified.^33^ Further details on the procedure^33,38^ and its application to the study of the dynamics of supramolecular polymers^25−27^ can be found in the original papers. Multiple infrequent WT-MetaD simulations^25^ were performed to activate and obtain the characteristic transition times for a jump-to-the-solvent event of a monomer from the tip of the fiber and for the event of the creation of a stacking defect in the backbone of each BTA fiber variant (see Figure 2). Similarly, the same infrequent WT-MetaD setup has been used to obtain the characteristic transition time scales for the same monomer exchange events using the minimalistic fiber CG models (see Figure 5). While it is worth underlining that all extracted transitions times are of little quantitative value—as these are extracted from approximated CG models—these can be still safely compared between them (*i.e.*, across the same CG models).^25−27^ The transition times obtained show the typical profile of a cumulative Poisson distribution, where the probability of observing at least one monomer exchange transition by time *t* is given by *P*_*n*≥1_ = 1 – e^–*t*/τ^, where τ is the characteristic transition time for each transition. We use this function to fit (Figure 5: colored Poissonian fits) the transition times distributions extracted from the WT-MetaD runs (Figure 5: in transparent gray) and to estimate the characteristic exchange time scales (τ). From these, we obtain the related average exchange rate constants as *k*_1_ = τ^–1^.

For the infrequent WT-MetaD runs activating the exchange-into-the-solvent events, we used as the collective variable (CV) the average number of contacts between the core of a monomer on the fiber tip (or on a bulk defect) and all other monomer cores in the fibers (excluding the solvophilic chains in the case of the minimalistic CG models), using the PLUMED variable “COORDINATIONNUMBER” (*R*_0_ = 0.5, *D*_MAX_ = 1.0). We used a HILLS height of 0.3 kcal mol^–1^, a HILLS width of 0.3, a deposition rate of one Gaussian every 5000 time steps, and a bias factor of 20. The same “COORDINATIONNUMBER” CV has been also used to estimate the characteristic transition times (τ) corresponding to the creation of a stacking defect in the bulk of BTA supramolecular fibers in organic solvent.

### Supervised Clustering Identification of Defects

Initially, to qualitatively identify the defects on each different CG fiber variant, we selected a set of PLUMED collective variables, computed for each individual monomer during the equilibrated CG-MD trajectories (last 20 μs of CG-MD). We used a total of 4 different variables: coordination (*R*_0_ = 0.67, *D*_MAX_ = 2.1) and minimum distance between central core CG beads, number of contacts between the core beads (*R*_0_ = 0.55, *D*_MAX_ = 2.1), and number of contacts between the charged beads composing the central dipoles (*R*_0_ = 0.38, *D*_MAX_ = 2.1).

All the data extracted from this heuristic analysis for each monomer in each fiber have been combined together and used as the input for a clustering algorithm, with the aim to separate the different populations (bulk monomers, tip monomers, and defects) and thus count the average number of defects in each different fiber. This clustering analysis has been performed using homemade python scripts that implemented the Spectral-clustering method.^34^ We set to 3 the number of clusters to be identified and used the nearest neighbors algorithm to construct the affinity matrix. To give a visual indication of how much the different clusters are populated in each case, we reported density plots for the three fibers in Figure S5. The script used for the supervised clustering is available at: www.github.com/GMPavanLab/Controlling-Exchange-Pathways.

### Unsupervised Clustering of Defects and of Defect Dynamics

In order to obtain a more complete characterization of the defects, their nature, and their dynamics, we turned to a more advanced unsupervised clustering approach. The potential of such an unsupervised machine learning analysis to automatically identify defects, and to explore defect dynamics in supramolecular polymers, has been recently described in detail in ref (29). This analysis used snapshots extracted from the equilibrated phase CG-MD trajectories for the 3 fibers (we used 10 μs with a Δ*t* = 10 ns temporal stride for each model). The displacement/order in the monomer cores at each CG-MD snapshot was analyzed by means of smooth overlap of atomic positions (SOAP),^35^ an agnostic and robust descriptor which provides a high-dimensional representation of the atomic/molecular environment surrounding each core in the systems. In order to capture the structural dynamicity in terms of structural/dynamic reorganization of the monomers in the three fiber models during the MD simulations (monomers’ reshuffling/reorganization), a SOAP vector was placed in the center of each monomer in the simulated fibers (*i.e.*, in the central bead containing the dipole), and we considered in the analysis all other monomer centers in the assemblies. Such a setup was previously shown to be well suited to capture the supramolecular dynamics (*i.e.*, monomers’ reshuffling and reorganization within the assembly), and to retain rich enough information on all possible structural states visited by the cores in the stacks during the simulations.^29^ These analyses were carried out using the python package Dscribe,^44^ setting parameters rcut = 8 Å, nmax = 8, lmax = 8 and leaving the rest as default, which proved to be a good setup to analyze similar systems as reported earlier.^29^ According to these parameters, the resulting output SOAP features space was 324-dimensional. Such a high-dimensional, rich output was then reduced *via* principal components analysis (PCA) to 3 main components, allowing retaining up to ∼94% of the global complexity/variability in the entire data set constituted by the three fibers (Figure 4b: the PCAs are then projected for visualization in 2D on the first two PCA dimensions, PCA1 and PCA2). This allowed us to ease the data treatment without losing too much accuracy (more than 90% of the information was retained in all 3 cases), and to retrieve from the cluster density profiles the low dimensional free-energy profiles (FES) of Figure 4c.^29^ Linear PCA dimensionality reduction was carried out using the python packaged Scikit-Learn.^45^ Finally, a density-based clustering scheme called Probabilistic Analysis of Molecular Motifs (PAMM)^36,46^ was applied. This allowed us to classify all accessible structural motifs in the fibers into micro-clusters, and hierarchically into macro-clusters, and to distinguish them by using different colors as shown in Figure 4. The parameters employed for the clustering calculations of the three data sets were all kept the same, fspread = 0.30, quick-shift = 1, bootstrap-runs = 73, merger-threshold = 0.005, apart from the grid-size sample used for the density estimation which was 1000 points for Fiber **1** and 2000 points for Fibers **2** and **3**. To complete the analysis, as described in a previous work,^29^ we qualitatively estimate the frequency of exchange between the main states (clusters in Figure 4c) in the fiber along the studied trajectories (see Figures S2–S4), summarized in matrix form. This is done by considering the probabilities of a certain event to occur (*P*), estimated from the PAMM clustering outputs, the number of frames *N*_frames_, and the frames temporal stride *t*_stride_ of the MD trajectory. Given that the number of events (*i.e.*, transitions) that occurs in a trajectory are defined as , and the total simulation time is given by *t*_tot_ = *N*_frames_ × *t*_stride_, transitions rates, *R*^tr^, in *time*^–1^ units can be computed as
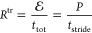
5The rates reported in Figure 4c are computed from the formula above.

## Supporting Information Available

Additional details of the molecular models and additional figures (PDF)
Movie 1: Unbiased simulation of Fiber **1** (MPG)
Movie 2: Unbiased simulation of Fiber **2** (MPG)
Movie 3: Unbiased simulation of Fiber **3** (MPG)

## Supplementary Material

nn1c01398_si_001.pdf

nn1c01398_si_002.mpg

nn1c01398_si_003.mpg

nn1c01398_si_004.mpg
