# Supplementary material for: Controlling Exchange Pathways in Dynamic Supramolecular Polymers by Controlling Defects
Source: ACS Nano. 2021 Sep 2;15(9):14229–41. doi: 10.1021/acsnano.1c01398 (PMC8482751; doi:10.1021/acsnano.1c01398)
Supplement: Supplementary file 1 — nn1c01398_si_001.pdf [file nn1c01398_si_001.pdf]

# Supporting Information for: Controlling Exchange Pathways in Dynamic Supramolecular Polymers by Controlling Defects

Anna L. de Marco,[†,‡] Davide Bochicchio,[†,‡] Andrea Gardin,[¶] Giovanni Doni,[†] and Giovanni M. Pavan[*,†,¶]

†*Department of Innovative Technologies, University of Applied Sciences and Arts of Southern Switzerland, Galleria 2, Via Cantonale 2c, CH-6928 Manno, Switzerland*

‡*Department of Physics, Università degli studi di Genova, Via Dodecaneso 33, 16100 Genova, Italy*

¶*Department of Applied Science and Technology, Politecnico di Torino, Corso Duca degli Abruzzi 24, 10129 Torino, Italy*

E-mail: giovanni.pavan@polito.it

## Supporting figures

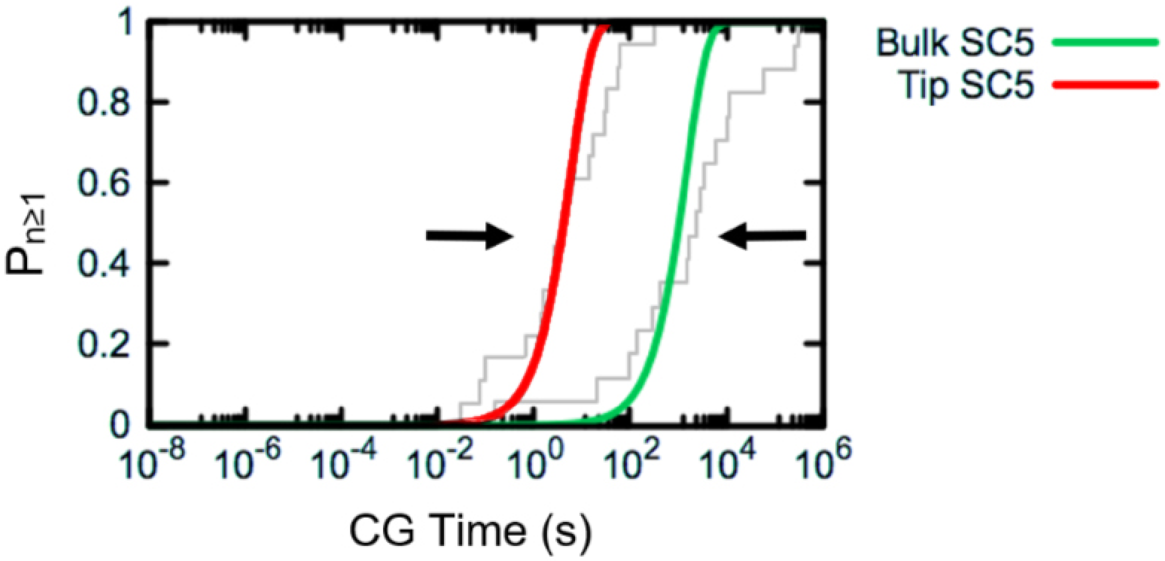


Figure S1: Exchange pathways of BTA supramolecular polymers in organic solvent. Cumulative Poisson probability distribution for the rare events of monomer exchange from the fiber tip (in red) and of generation of a bulk defect along the fiber (in green). These distributions refer to a modified BTA fiber in octane with increased solvophobicity (SC5 Martini beads instead of SC1). In such a case, the non-directional interactions between the monomers are effectively increased and the characteristic timescales for the creation of defects and exchange from fiber tips become closer to each other.

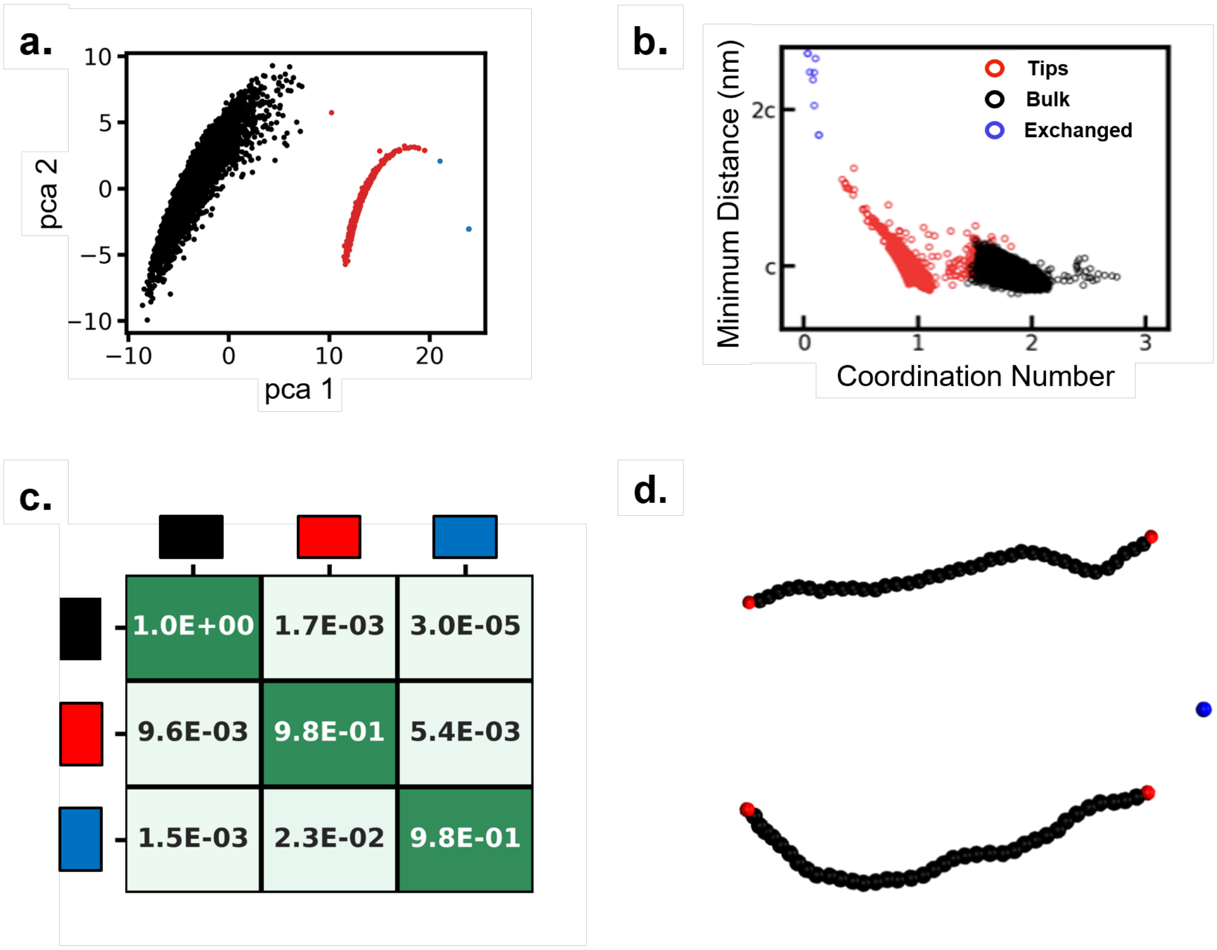


Figure S2: Clustering analysis for Fiber 1. a) Projection of PAMM clusters on two first principal components of SOAP vectors showing Fiber 1 complete structural motifs. The PCA model was trained using only Fiber 1 data set. b) Supervised clustering analysis of molecular motifs of Fiber 1. c) Interconversion probability matrices for the residence and exchange of monomers between the structural states, calculated directly from the PAMM analysis of the CG-MD trajectories. d) Fiber 1 CG-MD trajectory snapshots showing only the monomers core used for the analysis, colored according to the PAMM clusters.

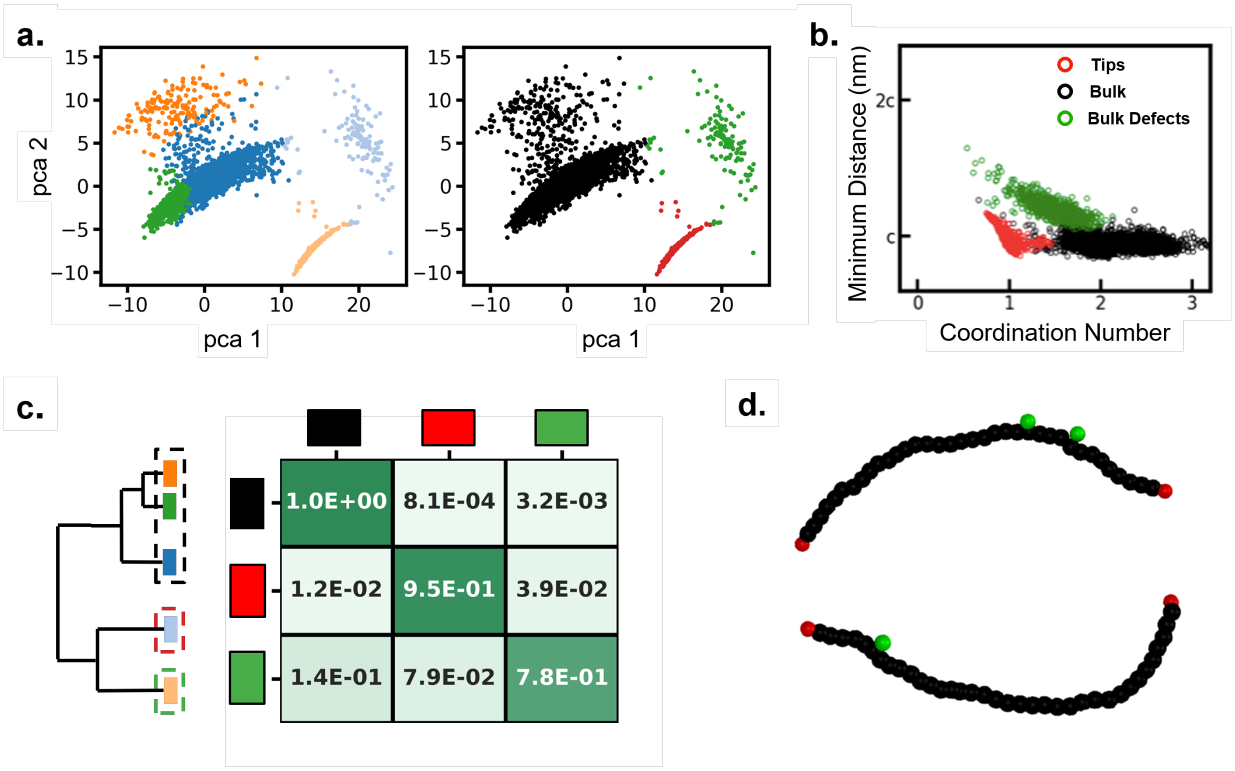


Figure S3: Clustering analysis for Fiber 2. a) Projection of PAMM micro- and macro-clusters (respectively left and right plot) on two first principal components of SOAP vectors showing Fiber 2 complete structural motifs. The PCA model was trained using only Fiber 2 data set. b) Supervised clustering analysis of molecular motifs of Fiber 2. c) Interconversion probability matrices for the residence and exchange of monomers between the micro- and macro-states, calculated directly from the PAMM analysis of the CG-MD trajectories. The dendrogram shows the hierarchical pairing used to build macro-clusters. d) Fiber 2 CG-MD trajectory snapshots showing only the monomers core used for the analysis, colored according to the PAMM macro-clusters.

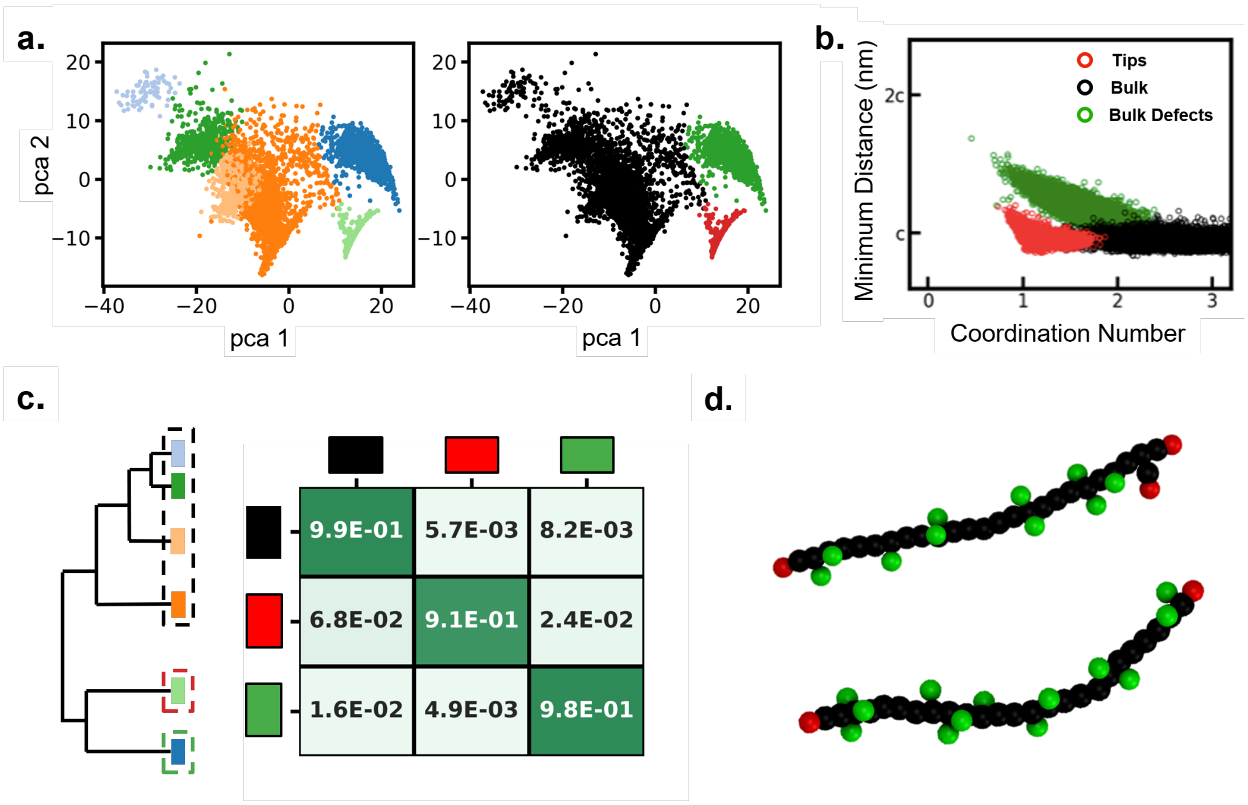


Figure S4: Clustering analysis for Fiber 3. a) Projection of PAMM micro- and macro-clusters (respectively left and right plot) on two first principal components of SOAP vectors showing Fiber 3 complete structural motifs. The PCA model was trained using only Fiber 3 data set. b) Supervised clustering analysis of molecular motifs of Fiber 3. c) Interconversion probability matrices for the residence and exchange of monomers between the micro- and macro-states, calculated directly from the PAMM analysis of the CG-MD trajectories. The dendrogram shows the hierarchical pairing used to build macro-clusters. d) Fiber 3 CG-MD trajectory snapshots showing only the monomers core used for the analysis, colored according to the PAMM macro-clusters.

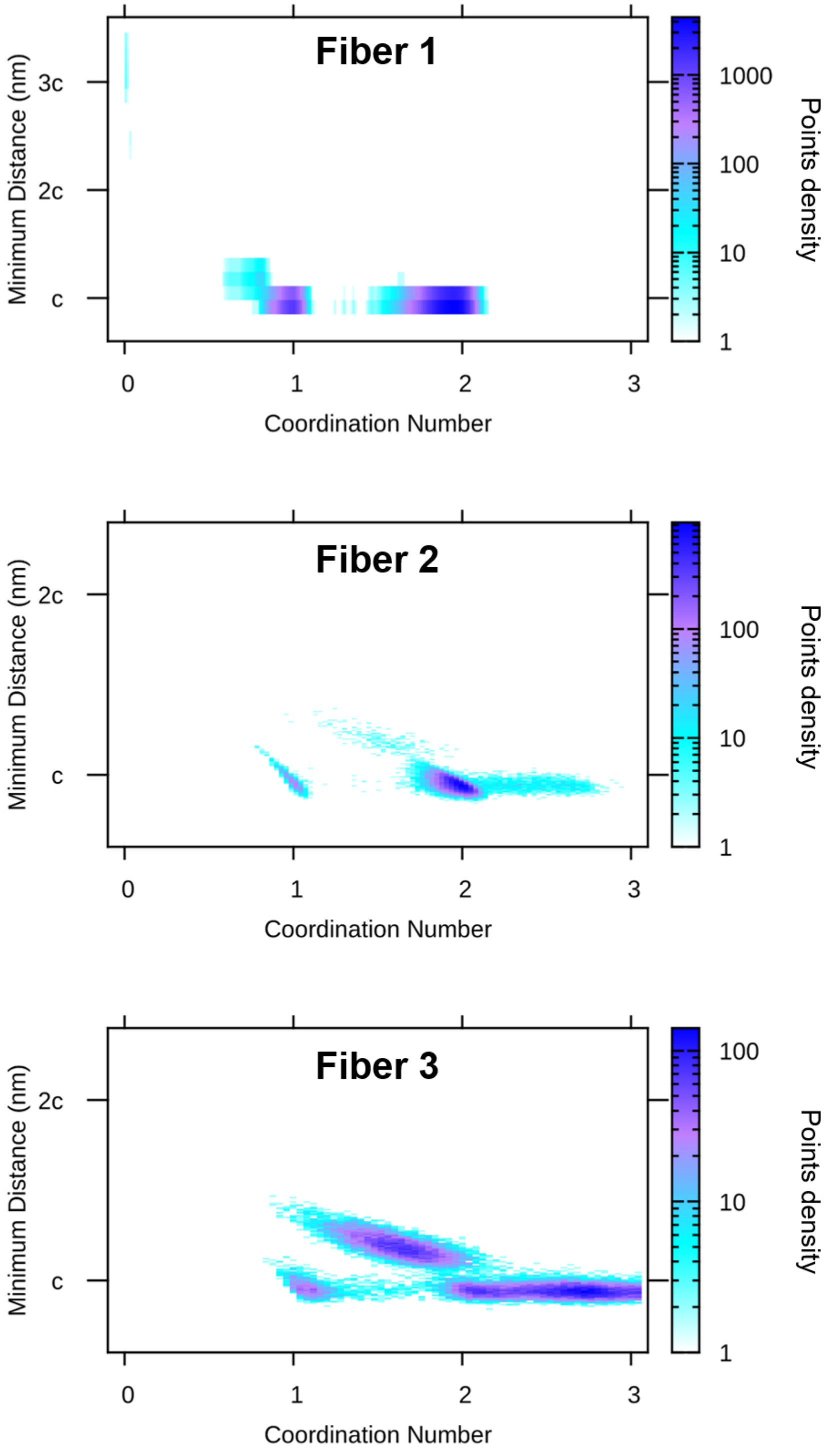


Figure S5: Density plots corresponding to the supervised analysis of Figure 3. For each fiber, we report the local density of points (logarithmic scale) in the Coordination-Minimum Distance projection.

## Topologies of the monomers

### Fiber 1:

*[moleculetype]*

; molname nrexcl

fib1 1

*[atoms]*

; id type resnr residu atom cgnr charge

1 C1 1 BENZ M1 1 0

2 C1 1 BENZ M2 2 0

3 C1 1 BENZ M3 3 0

4 C1 2 MID M4 4 0

5 C1 2 MID M5 5 0

6 C1 2 MID M6 6 0

7 C1 2 ALKA C7 7 0

8 C1 2 ALKA C8 8 0

9 C1 2 ALKA C9 9 0

10 C1 3 ALKA C10 10 0

11 C1 3 ALKA C11 11 0

12 C1 3 ALKA C12 12 0

13 C1 3 ALKA C13 13 0

14 C1 3 ALKA C14 14 0

15 C1 3 ALKA C15 15 0

16 P5 4 DIP D16 16 0

17 D 5 HP H17 17 1.45

18 D 5 HN H18 18 -1.45

*[constraints]*

16 17 1 0.14 22000

16 18 1 0.14 22000

*[bonds]*

; i j funct length

1 2 1 0.46 22000

2 3 1 0.46 22000

3 1 1 0.46 22000

1 16 1 0.2656 22000

2 16 1 0.2656 22000

3 16 1 0.2656 22000

1 4 1 0.46 5500

2 5 1 0.46 5000

3 6 1 0.46 5500

4 7 1 0.46 5500

5 8 1 0.46 5500

6 9 1 0.46 5500

7 10 1 0.46 5500

8 11 1 0.46 5500

9 12 1 0.46 5500

10 13 1 0.46 5500

11 14 1 0.46 5500

12 15 1 0.46 5500

*[angles]*

; i j k funct angle force.c.

3 2 5 2 150 1500

2 1 4 2 150 1500

1 3 6 2 150 1500

1 2 5 2 150 1500

3 1 4 2 150 1500

2 3 6 2 150 1500

1 4 7 2 180.0 10

2 5 8 2 180.0 10

3 6 9 2 180.0 10

4 7 10 2 180.0 10

5 8 11 2 180.0 10

6 9 12 2 180.0 10

7 10 13 2 180.0 10

8 11 14 2 180.0 10

9 12 15 2 180.0 10

17 16 18 2 180.0 1500

*[exclusions]*

17 18

**Fiber 2** - everything like fiber 1, except that the first 6 beads:

1 C5 1 BENZ M1 1 0

2 C5 1 BENZ M2 2 0

3 C5 1 BENZ M3 3 0

4 C5 2 MID M4 4 0

5 C5 2 MID M5 5 0

6 C5 2 MID M6 6 0

**Fiber 3** - everything like fiber 1, except that the first 6 beads:

1 N0 1 BENZ M1 1 0

2 N0 1 BENZ M2 2 0

3 N0 1 BENZ M3 3 0

4 N0 2 MID M4 4 0

5 N0 2 MID M5 5 0

6 N0 2 MID M6 6 0
